# *REM* genes controlling phyllotaxis and yield: bridging findings from *Arabidopsis thaliana* to *Brassica napus*

**DOI:** 10.3389/fpls.2025.1743387

**Published:** 2026-02-02

**Authors:** Carlotta C. Ferrario, Francesca Caselli, Shokhsanam Davlatboeva, Evert-Jan Blom, Arjen van Tunen, Veronica Gregis, Martin M. Kater

**Affiliations:** 1 Department of Biosciences, University of Milan, Milan, Italy; 2 Keygene Company, Wageningen, Gelderland, Netherlands

**Keywords:** *Arabidopsis thaliana*, *Brassica napus*, inflorescence architecture, REproductive Meristem (REM), seed yield

## Abstract

One of the main challenges in agriculture is the increasing demand to enhance plant yield per hectare. This is crucial not only for boosting food production to support a growing global population but also for minimizing land use for agricultural purposes. Seed yield is of particular importance since seeds are the primary source of carbohydrates and oil. The number of seeds that develop per plant is influenced by various factors, among which inflorescence architecture is a key trait. The geometrical organization of the inflorescence, known as phyllotaxis, plays an important role during reproductive development across many species. Despite its significance, the molecular mechanisms underlying phyllotactic patterning are still not fully understood. Recently, we demonstrated that the *REM* genes *AtREM34* and *AtREM35* are important in establishing phyllotactic patterns in Arabidopsis inflorescences. In this study, we investigated the genetic relationship between these two *REM* transcription factor genes and a closely related member, *AtREM36*. Interestingly, we show that double mutants of these genes display an increased number of siliques produced on the main stem. To explore the translational potential of this finding in the economically important seed crop *Brassica napus* (rapeseed), we identified and functionally analyzed, through complementation tests, a set of homologous *BnaREM* genes. This application-driven study uncovers novel genes associated with phyllotaxis and yield in the Brassicaceae family, contributing to our understanding of plant architecture and offering insights into sustainable strategies for crop improvement.

## Introduction

The world population is steadily increasing and is expected to reach 10 billion people by 2060 (Ritchie et al., 2024). This, along with the anticipated rise in meat and dairy consumption (Henchion et al., 2021) underscores the need to enhance crop production (Schwarz et al., 2020). However, this must be accomplished without expanding agricultural land to preserve ecosystems and avoid further greenhouse gas emissions associated with agricultural practices.

A strategy for yield improvement is increasing the number of fruits and seeds that a plant develops. This trait is determined by the architecture of the inflorescence, including its size, branching potential, and phyllotaxis — the geometrical arrangement of organs around the stem.

Phyllotaxis is regulated by the spatial and temporal emergence of primordia from the inflorescence meristem (IM). The divergence angle between primordia plays a crucial role in determining their spacing and, consequently, the potential number of primordia from which branches and flowers develop.

Different phyllotactic patterns can be observed in nature, among which the most common is described by the Fibonacci spiral. Observable in different model dicots, such as tobacco, tomato and Arabidopsis (Reinhardt, 2005) it is characterized by consecutive angles which diverge from each other by 137.5°, the so-called “golden angle”. This precise patterning has attracted the attention of mathematicians, physicists and biologists for centuries, who developed models and theories trying to explain such a regularity (Adler et al., 1997; Okabe, 2016; Barabé and Lacroix, 2020; Yin, 2021).

In plant biology, the current model identifies the phytohormone auxin as the primary regulator of phyllotaxis. Indeed, in the inflorescence meristem, the initiation of the primordium is triggered by this hormone. Furthermore, the primordium acts as an auxin sink, thereby depleting auxin in the surrounding area and creating a zone where organogenesis is inhibited (Traas, 2013; Galvan-Ampudia et al., 2016; Yin, 2021).

The spatial distribution of auxin depends both on its biosynthesis and transport, and in Arabidopsis it has been shown that mutants in genes related to such processes have a defective phyllotactic pattern or can’t develop primordia at all (Okada et al., 1991; Bennett et al., 1995; Okal et al., 1999; Reinhardt et al., 2003; Heisler et al., 2005; Prasad et al., 2011; Pinon et al., 2013; Bhatia et al., 2016; Shi and Vernoux, 2019; Yin, 2021). Moreover, similar mutated patterns can be observed in mutants where auxin is correctly distributed but not perceived, as observed in some *auxin response factor (arf)* mutants (Przemeck et al., 1996; Sessions et al., 1997; Nemhauser et al., 2000; Simonini et al., 2017; Zhang et al., 2022).

Interestingly, phyllotaxis stability is linked to inflorescence meristem dimensions: both natural variations in IM size among different ecotypes and the environmental-driven increase of meristem size by reducing day length led to permutations in the phyllotactic pattern (Landrein et al., 2015).

Furthermore, phyllotactic patterning and IM dimensions have been reported to correlate with the number of siliques that a plant will develop. In Arabidopsis, for instance, the characterization of mutants such as *ckx3 ckx5* (Bartrina et al., 2011), *arf3* (Zhang et al., 2022) and plants carrying a miR172 resistant *AP2* allele (Sang et al., 2022) display simultaneous variations in meristem dimensions, phyllotactic patterning and increased yield, supporting the relation among these three phenotypes. Relevantly, in *arf3* and *ckx3 ckx*5 mutants, the important hormonal pathways of auxin or cytokinin are involved.

Even if the direct link is still to be elucidated, the common correlation between these traits suggests that altering inflorescence features such as phyllotaxis and meristem dimension might be promising strategies to increase crop yield.

Recently, we have characterized two Arabidopsis mutants in genes belonging to the *REproductive Meristem* (*REM*) family, which display altered phyllotaxis and an increased inflorescence meristem (IM) area (Caselli et al., 2025). AtREM34 and AtREM35 control phyllotaxis by interacting with AtARF7 and AtARF19, evidencing a link between the REM TFs and the auxin pathway.

REM transcription factors belong to the B3 DNA binding domain superfamily together with the ARF (Auxin Response Factor), ABI3/VP1 (ABscisic acid Insensitive3/Viviparous1), HIS (High-level expression of Sugar Inducible gene), and RAV (Related to Abi3/Vp1) families (Swaminathan et al., 2008; Romanel et al., 2009). The Arabidopsis genome encodes 87 B3 proteins, of which 45 are classified as REM family members, making it the most numerous and divergent family within this superfamily (Romanel et al., 2009). Phylogenetically, the REM genes can be divided into subclasses based on specific domains they contain (Romanel et al., 2009).

Despite being the most abundant, the *REM* genes are the least characterized among the B3 superfamily. Nevertheless, recent studies have been uncovering their involvement in several developmental processes related to the regulation of flowering time, inflorescence and gametes development (Caselli et al., 2025; Gómez-Mena et al., 2005; Matias-Hernandez et al., 2010; Heo et al., 2012; Mendes et al., 2016; Richter et al., 2019; Yu et al., 2020; Li et al., 2021; Wu et al., 2021; Manrique et al., 2023; Levy et al., 2002).

Mantegazza et al. (2014), identified a cluster of *REM* genes —*AtREM34*, *AtREM35*, and *AtREM36* – which was considered particularly of interest for studying its potential role in Arabidopsis flower and inflorescence development because of the expression profiles of the three genes and their putative regulators. They are expressed in the inflorescence and floral meristems (Mantegazza et al., 2014), as well as in the petals and reproductive organs at different stages of development (Caselli et al., 2019). They are putative direct targets of MADS-domain transcription factors that control flower development, such as AP1 (Kaufmann et al., 2009), AP3 and PI (Wuest et al., 2012), and SVP (Gregis et al., 2013).

In Arabidopsis, *AtREM34*, *AtREM35*, and *AtREM36* are located on chromosome 4 and arranged in tandem within a broader cluster of phylogenetically related *REM* genes, belonging to subclass IX. Nine out of the fifteen *REM IX* genes belong to this locus, while almost all the others are part of a duplication on chromosome 2 (Romanel et al., 2009; Mantegazza et al., 2014). The genes belonging to this subclass are thought to come from several tandem duplication events and therefore have the potential to be redundant. Specifically, *AtREM34*, *AtREM35*, *AtREM36* and *AtREM37* are closely related to each other; however, the latter is not expressed in Arabidopsis (Romanel et al., 2009; Mantegazza et al., 2014).

We have shown that *AtREM34* and *AtREM35* play multiple roles in plant development, including involvement in gametophyte development, determination of phyllotaxis, and regulation of Shoot Apical Meristem (SAM) and IM sizes (Caselli et al., 2019, 2025; Manrique et al., 2023). *AtREM34* is also involved in flowering time regulation (Manrique et al., 2023).

Since the phenotypes displayed by *atrem34* and *atrem35* single and double mutants might be relevant in the context of yield improvement, to deepen our knowledge of this cluster of *AtREM* genes, here we generated and characterized the *atrem36* single mutant and studied all possible double mutant combinations with *atrem34* and *atrem35.*

Notably, these double mutants are characterized by an aberrant phyllotaxis and an increased number of siliques on the primary inflorescence. This observation prompted us to further investigate the possibility of translating these results to the phylogenetically related crop species *Brassica napus* (rapeseed), which belongs, like *Arabidopsis thaliana*, to the Brassicaceae family.

*Brassica napus* is an allotetraploid, formed 7500 years ago from the interspecific hybridization between *Brassica rapa* and *Brassica oleracea* (Chalhoub et al., 2014), and it is an important oil crop, producing approximately 11% of the world’s vegetable oil (FAO, 2025; 2022 dataset – with major processing by Our World in Data). Breeding for *Brassica napus* genotypes with an increased yield could therefore be of great economic value.

As a first step to explore the possibility of increasing seed yield by changing inflorescence architecture in *B. napus* based on the knowledge obtained for *REM* genes in Arabidopsis, we focused on the identification of Arabidopsis *AtREM34*, *AtREM35* and *AtREM36* orthologous genes in *B. napus.* Then, their potential functional conservation was investigated by interspecific complementation. Our data provide a promising starting point for yield improvement in *B. napus* and other Brassicaceae.

## Materials and methods

### Plant material and growth conditions

*Arabidopsis thaliana* Col-0 and *Brassica napus* plants were grown under long-day conditions, with 16h of light/8h dark, at 22°C.

*atrem34, atrem35* and *atrem34 atrem35* mutants were previously generated and described (Caselli et al., 2025). *rem36* was generated following the protocol published by Fauser et al. (2014). The protospacer sequence (GTGATCTGACGAAAAAAGGT) was selected with the aid of the CRISPR-P 2.0 software (http://crispr.hzau.edu.cn/CRISPR2). The resulting mutation is a T insertion which falls into the first B3 DNA binding domain, causing a frameshift and the formation of an early stop codon in position aa31 (**Supplementary Figure S1**). *atrem34 atrem36* and *atrem35 atrem36* double mutants were generated by re-transforming single mutants, as the in-linkage position of the genes makes it unlikely the generation of higher order mutants by crossing. Different transformation events always gave the same mutation.

### Phyllotactic pattern measurement and yield analysis

The phyllotactic pattern was measured as described before (Peaucelle et al., 2007) on the main inflorescence of 2-month-old plants using a 3D clockwise protractor. The first 3 cm of the stem was not considered because elongation is incomplete in this region. The divergence angle was measured by considering the insertion point of the two successive floral pedicels. The clockwise or anticlockwise orientation of the phyllotaxis was determined by following the direction that gives the smallest average divergence angle.

The yield analysis was performed by counting the number of siliques produced on the main inflorescence of completely developed plants. The number of seeds per silique was counted in green but completely elongated siliques with the aid of a stereomicroscope.

Kruskal-Wallis and Dunn’s *post hoc* test were used to evaluate the significance of the results, according to the normality and homoscedasticity of the data (GraphPad Prism).

### Homology and phylogenetic analysis

The homologous sequence identification was performed by querying proteome databases from Ensembl plants using Jackhmmer (Eddy, 2011), and resultant sequences were filtered to retain only those with a minimum sequence identity of 40%. Specifically, for the *REM IX* clade homology analysis, all the REM IX *Arabidopsis thaliana* proteins were used to query the *Brassica napus* proteome. To narrow down and fine-tune the results, AtREM34, AtREM35, AtREM36 and AtREM37 were used to query *Arabidopsis lyrata*, *Brassica oleracea, Brassica rapa* and *Brassica napus* proteomes.

The homologous sequences obtained were aligned by Clustal Omega 1.2.4 (default parameters, Sievers et al., 2011), and their phylogenetic relationships were inferred by RAxML-NG (Kozlov et al., 2019) with the WAG+G substitution model (50 bootstrap replicates).

### RNA extraction and qRT-PCR

RNA was extracted from *Brassica napus* tissues using the LiCl method (Verwoerd et al., 1989). For each sample, 500 ng of RNA was retro-transcribed using iScript kit (BioRad). qRT-PCR assay was performed using iTaq Universal SYBR Green supermix (BioRad) in a Bio-Rad iCycler iQ Optical System (software version 3.0a). Three biological replicates, with three technical replicates for each sample, were analyzed.

Relative transcript enrichment of the targets of interest was calculated by normalizing the amount of mRNA against the *BnaACTIN-7* transcript (NCBI reference sequence NM_001316010.1). Expression of genes was calculated using the 2^−ΔCt^ method. The primers employed for qPCR are listed in **Supplementary Table S1**.

### In situ hybridization

IM-enriched tissues were fixed in FAA (50% ethanol, 5% acetic acid, 3.7% formaldehyde) under vacuum for 15 min, dehydrated in ethanol and bioclear (Bioptica) and embedded in Paraplast Plus (Sigma-Aldrich). *In situ* hybridization was performed as previously described (Coen et al., 1990) with slight modifications. Digoxigenin- labeled probes were synthesized with T7 RNA polymerase (Promega) and tested by dot blot.

The specificity of the signal was assessed by hybridizing the same type of tissues with the sense probe (**Supplementary Figure S5**).

Slides were imaged with the aid of a Zeiss Axiophot^®^ microscope equipped with differential interference contrast (DIC) optics.

Primers employed for probe synthesis are listed in **Supplementary Table S1**.

### Complementation test

The CDS of 4 of the *Brassica napus* candidate genes were cloned from *Brassica napus* cDNA samples in p207 pDONR vector by BP Gateway reaction and were subsequently cloned into the pDEST vector pB2GW7 by LR Gateway reaction.

The constructs were transformed into *Agrobacterium tumefaciens* GV3101 and subsequently into the homolog *Arabidopsis* mutants by floral dip.

The expression of the construct was tested in T1 inflorescences. RNA extraction and RT-qPCR were performed as described above. Relative transcript enrichment of the targets of interest was calculated by normalizing the amount of mRNA against the *AtRCE1* (At4g36800) transcript (Pribil et al., 2010). Expression of the genes was calculated using the 2^−ΔΔCt^ method, using the Wild Type as a normalizer.

The T2 generation was used for the phenotype complementation analysis. The phyllotactic pattern was analyzed and the percentage of angles falling in the 130°-150° range (range around the golden angle, according to the precision of the instrument used) was assessed to evaluate the phenotype complementation.

Parametric and non-paramentric analysis of variance and *post-hoc* tests were used to evaluate the statistical significance of the results. Specifically, according to the normality and homoscedasticity of the data, ANOVA and Dunnett’s test were used for the *atrem35* and *atrem36* complementation, and Kruskal Wallis and Dunn’s test for *rem34* one. The *post hoc* test was run versus the WT and the untransformed *rem*.

The primers used for cloning and for the expression analysis are listed in **Supplementary Table S1**.

### Yeast-2-hybrid

The coding sequences of the genes of interest were cloned in the GAL4 system Gateway vectors (pGADT7 and pGBKT7; Clontech Laboratories, Inc.) by LR Gateway reaction starting from the p207-derived entry vector mentioned above.

The Y2H constructs for AtARF19 and AtARF7 were already available in the lab (Caselli et al., 2025).

The pGADT7 and pGBKT7 vectors were transformed into AH109 and Y187 strains, respectively. Yeast transformation and mating were performed as described by de Folter and Immink, 2011.

The protein-protein interaction assays were performed on selective yeast synthetic dropout medium lacking Leu, Trp, His and Ade, supplemented with different concentrations of 3‐aminotriazole. Plates were grown for 3–5 days at 28 °C.

AtREM35-AtREM35 and AtREM34-AtREM34 were used respectively as positive and negative controls (Caselli et al., 2019).

### Bimolecular fluorescence complementation

The same CDSs used for Y2H were cloned into pYFPN43 and pYFPC43, and the resulting constructs were transformed into *Agrobacterium tumefaciens* GV3101. The interactions were tested by co-infiltrating the abaxial surface of *Nicotiana benthamiana* leaves with the viral suppressor p19K construct. After 3 days, interactions were visualized by Laser Scanning Confocal Microscope Nikon A1 (laser emission 488 nm, YFP fluorescence collection between 500 and 550 nm, chlorophyll autofluorescence collection 660–740 nm).

As positive control, AtREM35 homodimerization was tested, while AtREM34 homodimerization was used as negative control (Caselli et al., 2019), coupled with p19K only infiltration.

## Results

### The combination of AtREM34, AtREM35 and AtREM36 mutations leads to an increased siliques number on the main inflorescence axis

Recently, we showed that *AtREM34* and *AtREM35* control phyllotactic patterning in Arabidopsis (Caselli et al., 2025). Since *AtREM36* is closely related to *AtREM34* and *AtREM35* and is in linkage with them in the same gene cluster (Romanel et al., 2009), we generated, using the CRISPR-Cas9 system, the *atrem36* mutant. The mutant presents a T insertion into the first B3 DNA binding domain, which causes a frameshift and the formation of an early stop codon in position aa31. We also generated the *atrem34 atrem36* and *atrem35 atrem36* double mutants and investigated inflorescence architecture features in all these mutant combinations.

**Figure 1:**
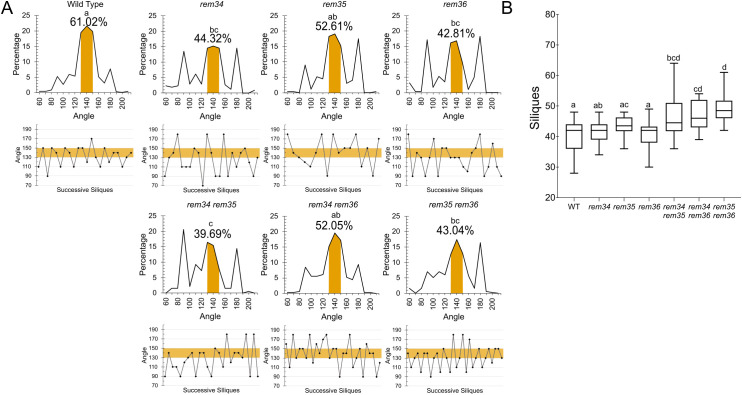
Inflorescence architecture analysis **(A)** Phyllotaxis patterns of the *atrem* single and double mutants. The top graphs show the distribution of angles between two successive siliques, the values of the divergence angle are indicated on the x-axis, while the percentage for each value is shown on the y-axis. The canonical angle range (130°-150°) is highlighted and the percentage of angles falling in that range is reported. Wild type (20, plants, 472 angles) *atrem34 (*17 plants, 487 angles), *atrem35* (12 plant, 268 angles), *atem36* (13 plant, 285 angles), *atrem34 atrem35* (9 plants, 194 siliques), *atrem34 atrem36* (12 plants, 342 angles), and *atrem35 atrem36* (11 plants, 316 angles). Statistical significance was calculated by ANOVA and Tukey *post hoc* test according to the normality and homoscedasticity of the data. Different letters are given to significantly different genotypes (p<0.05). The bottom graphs show a representative phyllotactic pattern of a single plant for each genotype; the canonical angle range (130°-150°) is highlighted. **(B)** Siliques on primary stem. Wild type n=30, *atem34* n=29, *atem35* n=30, *atem36* n=23, *atem34 atrem35* n=26, *atrem34 atrem36* n=29, and *atrem35 atrem36* n=21. Statistical significance was calculated by Kruskal-Wallis and Dunn’s *post hoc* test according to the normality and homoscedasticity of the data. Different letters are given to significantly different genotypes (p<0.05).

All single and double mutants showed divergence from the golden angle (137.5°), with angles around 90° or 180° (**Figure 1A**).

We also investigated these mutants for the impact on plant yield by counting the number of siliques on the main inflorescence. The primary inflorescence of wild type plants carries on average 40.65 siliques, similarly to what was registered for the single *atrem34, atrem35* and *atrem36* mutants. Interestingly, the double mutants produced on average a higher number of siliques: *atrem34 atrem35* 46.23 siliques; *atem34 atrem36* 46.59 siliques and *atrem35 atrem36* 49.25 siliques, accounting for approximately a 20% increase of siliques on the main stem (**Figure 1B**).

Importantly, since *AtREM34*, *AtREM35* and *AtREM36* are also expressed in the gametophyte, we counted the number of seeds per silique in all the double mutant combinations. Since all single and double mutant combinations develop, on average, the same number of seeds per silique as the wild type (**Supplementary Figure S2**), the mutations in the *AtREM* genes resulted in an increased siliques number without affecting seed development, thus ensuring an increase in total seed production on the main stem.

The observation of the phyllotactic phenotype for single and double mutants and the yield increase in double mutants suggests that the genes under study have a redundant function in the control of inflorescence architecture.

### Identification of REM34, REM35 and REM36 homologs in Brassica napus

Both *Brassica napus* and *Arabidopsis thaliana* belong to the Brassicaceae family. Since increasing seed yield per hectare is a major breeding goal in *B. napus*, we have chosen to evaluate the potential for translating our findings from Arabidopsis to this valuable crop.

To identify the *B. napus* genes orthologous to *AtREM34*, *AtREM35* and *AtREM36*, the phylogenetic conservation of the *REM* genes in *B. napus* was investigated.

*AtREM34*, *AtREM35* and *AtREM36* genes are located in tandem on chromosome 4, within a larger cluster of *REM* genes which are part of the subclass IX. *AtREM37* is in linkage with our genes of interest and clusters in the same phylogenetic group (Romanel et al., 2009). These observations suggest a possible redundancy of *AtREM37* with *AtREM34*, *AtREM35* and *AtREM36.* However, the gene was not characterized in Arabidopsis because it is poorly expressed (Mantegazza et al., 2014). Due to the high redundancy and duplication level observed in the *REM* family, identifying all high-confidence redundant genes is of paramount importance. Therefore, our research was broadened to include also the *B. napus REM37* homologs.

**Figure 2:**
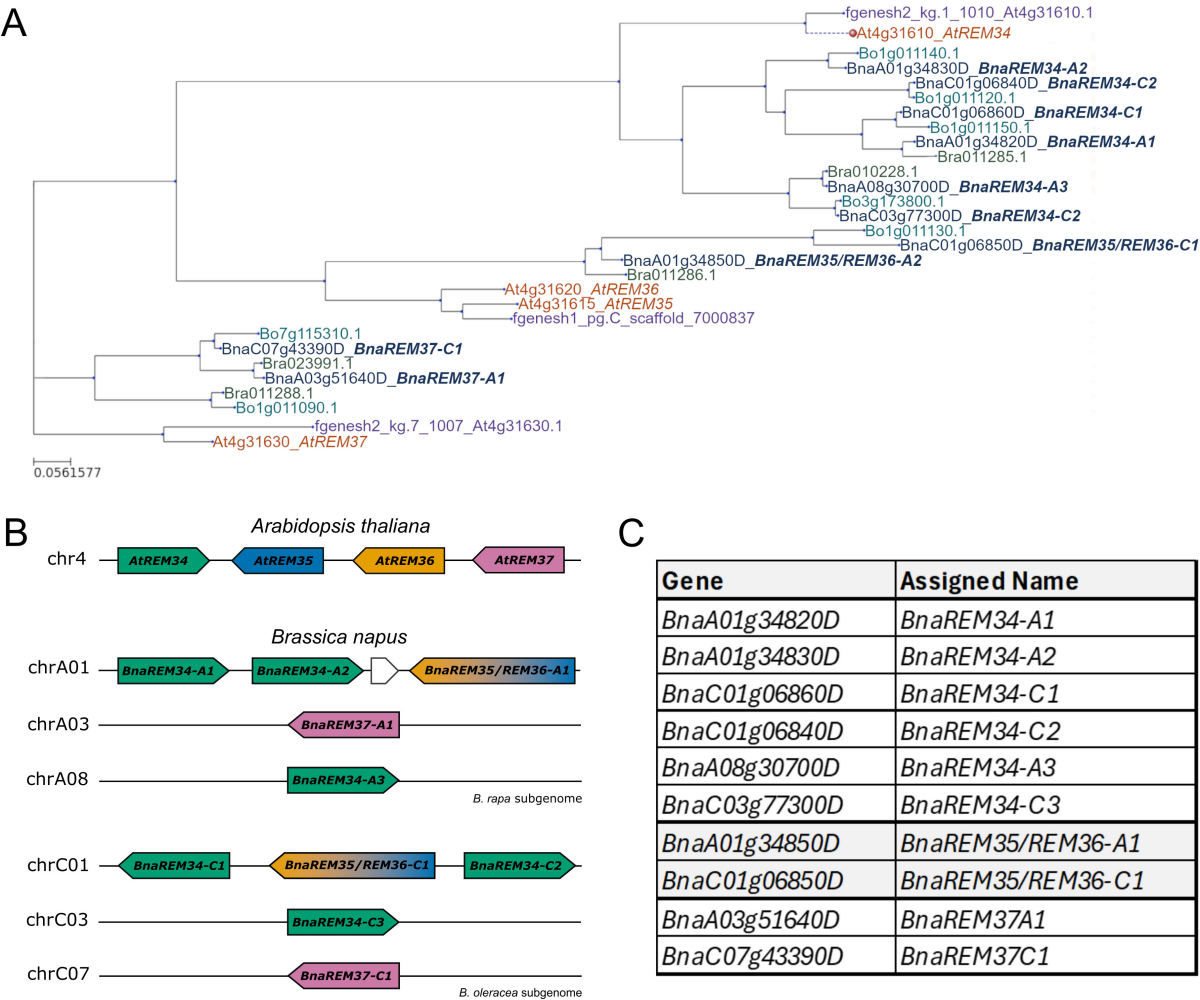
REM34, REM35, REM36 and REM37 *Brassica napus* homologs. **(A)** Phylogeny of the *Arabidopsis thaliana* (orange) *Brassica napus* (blue), *Brassica oleracea* (teal), *Brassica rapa* (green), and *Arabidopsis lyrata* (purple) proteins, providing a comprehensive analysis. Distance as amino acid substitution per site. **(B)**. Chromosome location of the genes of interest in *Arabidopsis thaliana* and *Brassica napus*. Homologs are indicated with the same color in the two genomes **(C)** Table indicating *Brassica napus* genes and the corresponding name assigned in this work.

The protein sequence of the Arabidopsis AtREM34, AtREM35, AtREM36 and AtREM37 were used to query *Arabidopsis lyrata*, *Brassica oleracea*, *Brassica rapa* and *Brassica napus* proteomes from Ensembl plants. The parental diploid species (*Brassica rapa* and *Brassica oleracea*) were included to provide additional soundness to the results (**Figure 2A**).

To confirm our results and minimize the risk of misassigning the closest homologs, we queried the *B. napus* proteome using the entire Arabidopsis REM IX family (REM28-REM42, according to Romanel et al., 2009, **Supplementary Figure S3**).

These analyses allowed the identification of six *B. napus* REM34 candidate homologs, two REM37 candidate homologs and two proteins which are closely related to both AtREM35 and AtREM36 (**Figures 2A, B**). To simplify the nomenclature, we named the *B. napus* REM with a number corresponding to the REM homolog in *Arabidopsis*, followed by “A” or “C” to indicate the *B. rapa* (AA) or *B. oleracea* (CC) subgenome, and a successive number (**Figure 2C**).

From our phylogenetic analysis we could not univocally assign two *B. napus* genes, *BnaREM35/REM36-A1* and *BnaREM35/REM36-C1*, to one Arabidopsis gene. This might be linked to the fact that *AtREM35* and *AtREM36* might have duplicated in Arabidopsis after the division of the Brassica-Arabidopsis clades. Indeed, the estimated age of duplication for *AtREM35* and *AtREM36* is 14 million years ago (Romanel et al., 2009), which is close to the timing of the *Brassica*-*Arabidopsis* clade split (approximately 14–24 million years ago; Bowers et al., 2003; Lysak et al., 2005; Wang et al., 2011).

Further analysis revealed the chromosome positioning of the *B. napus* homologs (**Figure 2B**). The linkage context observed in Arabidopsis is at least partially conserved in both diploid subgenomes, with two *BnaREM34* homologs and the homolog of *REM35/REM36*, which are located in linkage on chromosome A1 in the *Brassica rapa* subgenome (*BnaREM34-A1*, *BnaREM34-A2*, *BnaREM35/REM36-A1*) and on chromosome C1 of the *Brassica oleracea* subgenome (*BnaREM34-C1*, *BnaREM35/REM36-C1*, *BnaREM34-C2*).

On the other hand, the third *REM34* homolog, as well as *REM37* homolog, are in different genomic positions in both the parental subgenomes (specifically, *BnaREM34-A3* is located on chromosome A08, *BnaREM34-C3* on chromosome C03, *BnaREM37-A1* on chromosome A03 and *BnaREM37-C1* on chromosome C07). This genomic landscape is coherent with the previous collinearity studies of Delourme et al., 2013.

The homologous *B. napus* REM proteins have high sequence similarity with the Arabidopsis counterparts. Indeed, all *B. napus* homologs have more than 60% of protein similarity with their Arabidopsis relative, apart from *BnaREM35/REM36-C1* which shows a slightly lower protein similarity (**Supplementary Figure S4**).

The homologous of the same AtREM have high sequence similarity between themselves too. The BnaREM34 proteins display between 70% and 91% similarity, the BnaREM37 proteins show 80% similarity, and BnaREM35/REM36A1 and BnaREM35/REM36-C1 diverged more, having 68% protein similarity.

Between BnaREM34 homologs, the homeologs of the two subgenomes show the minor divergence (e.g. BnaREM34-A1 and BnaREM34-C1, BnaREM34-A2 and BnaREM34-C2, BnaREM34-A3 and BnaREM34-C3) (**Supplementary Figure S4**). This is expected in recent hybridization between related species as it is in the case of *B. napus* (Chalhoub et al., 2014).

### Conserved expression patterns of BnaREM genes

The expression profiles of the *BnaREM* genes of interest for this study were investigated by qRT-PCR, in vegetative tissues, such as roots, cotyledons, leaves, hypocotyles, SAM-enriched apexes, and in reproductive tissues: inflorescence apices (containing the inflorescence meristem and early developing flowers), developed flowers and young siliques.

**Figure 3:**
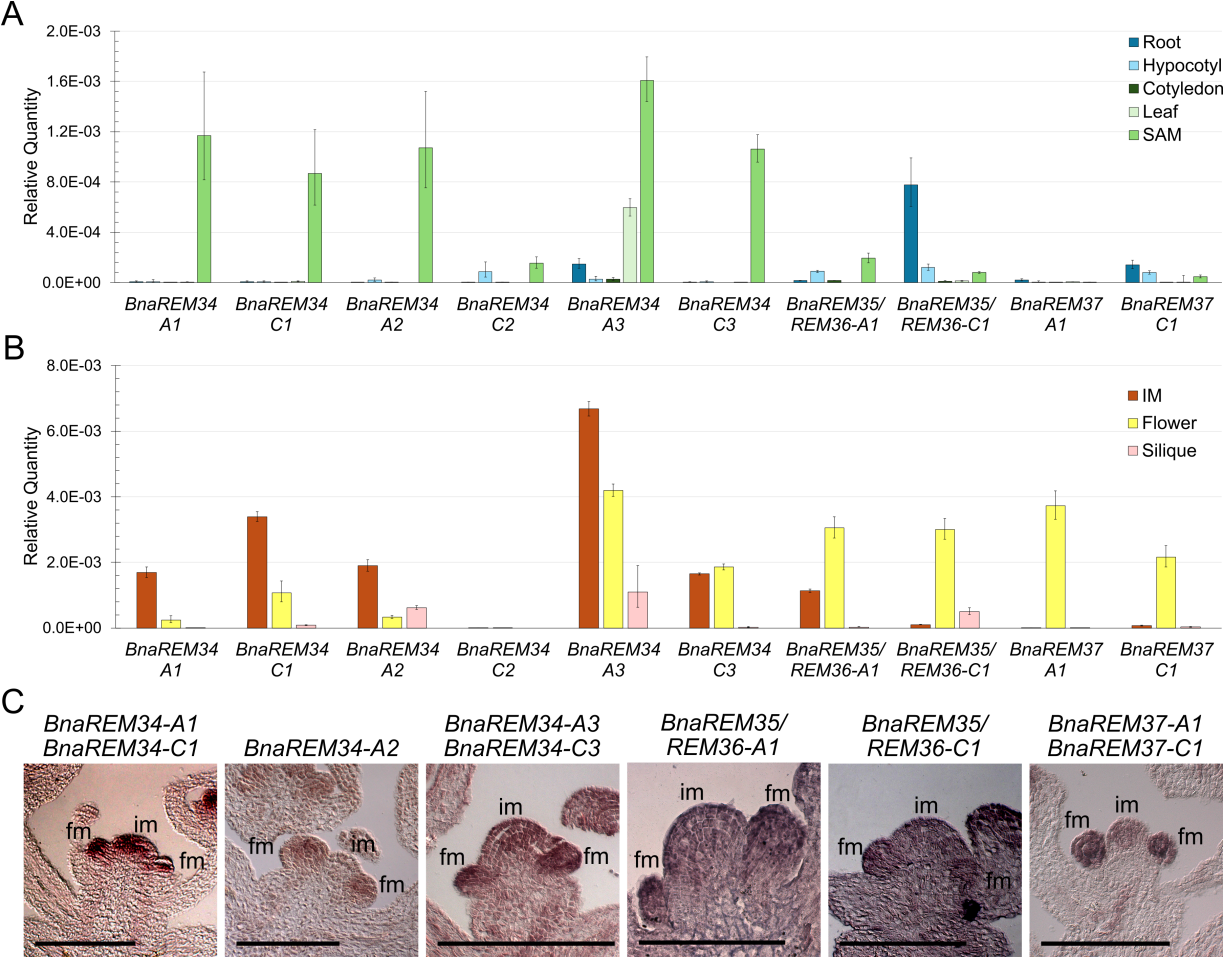
*BnaREMs* expression analysis. **(A)***BnaREM* genes expression in vegetative tissues: root, hypocotyl, cotyledons, leaf and Shoot Apical Meristem (SAM). The graphs show one representative biological replica out of 3. The fold change is calculated compared to the housekeeping gene *BnaACTIN-7* by the 2^-ΔCt^ method. **(B)***BnaREM* genes expression in reproductive tissues: Inflorescence Meristem (IM), flower and silique. The graphs show one representative biological replica out of 3. The fold change is calculated compared to the housekeeping gene *BnaACTIN-7* by the 2^-ΔCt^ method. **(C)**. *In situ* hybridizations. Given the probe length and the high CDS sequence identity of the genes under study, each probe targets the homeologs of the two diploid progenitors, except for *the REM35/REM36* genes, which diverged more. The *BnaREM34*, *BnaREM35/REM36* and *BnaREM37* homologs are all expressed in the floral meristems (fm) and in the inflorescence meristem (im). Scale bars 200 µm. Sense probe controls are shown in **Supplementary Figure S5**.

The *BnaREM34* homologs show a similar expression pattern as the At*REM34* gene, being expressed in the shoot apical meristem-enriched samples, inflorescence apex and in flowers, except for *BnaREM34-C2*, which is lowly expressed in all the tested tissues. However, *BnaREM34-A3* displays a broader expression profile compared to the others, being also present in leaves and siliques (**Figures 3A, B**). The *BnaREM35/REM36* and *BnaREM37* homologs exhibited a general lower expression in all the analyzed tissues and a slightly different profile compared to *BnaREM34s*, indeed they all show the highest expression in floral tissues and not in the inflorescence meristem. *BnaREM35/REM36-A1* was also expressed in the inflorescence meristem (IM) and, to a lesser extent, in the shoot apical meristem (SAM) – enriched samples. *BnaREM35/REM36-C1* was expressed in roots and siliques. Finally, *BnaREM37* genes were expressed in flowers (**Figures 3A, B**). In general, the expression of the tested *BnaREM* genes is predominantly present in reproductive structures.

The spatial expression profile of the *BnaREM* genes was further investigated throughout the reproductive phases by *in situ* hybridization analysis using *B. napus* inflorescences.

For this purpose, given the length of the probe and the high CDS sequence identity of the homeologs, each probe targets the homeologs of the two diploid progenitors, except for the *BnaREM35/REM36* genes, which divergence allowed gene-specific probes design.

For all the genes, the signals were mainly localized in the floral meristems and/or in the inflorescence meristem dome, which is compatible with a role in phyllotaxis determination (**Figure 3C**).

We additionally analyzed their expression during later stages of floral development. Across floral development, the signal was localized in petals, anthers, carpels and nectary glands, but it was excluded from sepals. Later, the expression was also clearly visible in the ovules (**Supplementary Figure S5**).

The expression patterns of all the *BnaREM* genes are highly similar to those of the Arabidopsis homologs (Franco-Zorrilla et al., 2002; Mantegazza et al., 2014; Caselli et al., 2019), suggesting a possible conservation of their biological role.

Additionally, in contrast to what was reported in Arabidopsis, *BnaREM37* expression profile was found to overlap with the expression of the other *REM* genes under study, suggesting a possible redundancy between these genes.

### Functional conservation of BnaREM genes in the regulation of phyllotaxis

Since the expression patterns of the *BnaREM* genes are compatible with a function in the determination of phyllotaxis we explored their functional conservation by complementation tests using the Arabidopsis *atrem*34, *atrem35* and *atrem36* single mutants, evaluating the percentage of angles falling into the range of the golden angle (130-150° range).

Since the homologous genes of the *B. napus A* and C subgenomes encode REM proteins with very high percentages of similarity (**Supplementary Figure S4**), and because the allopolyploidization is recent, we focused on the genes derived from the A subgenome, originating from *Brassica rapa*. We therefore cloned *BnaREM34-A1*, *BnaREM34-A3*, *BnaREM35/REM36-A1*, and *BnaREM37-A1* under the control of the 35S constitutive promoter. We excluded BnaREM34-A2 since its expression pattern and protein sequences closely resemble those of BnaREM34-A1 (**Figure 2**, **Supplementary Figure S4**).

The *BnaREM34-A1* and *BnaREM34-A3* constructs were used to transform the *atrem34* mutant, while the *BnaREM35/REM36-A1* construct was used to transform both the *atrem35* and *atrem36* single mutants. Since we couldn’t evaluate the functional conservation of *BnaREM37* due to the absence of a corresponding functional mutant in Arabidopsis, we decided to transform the *BnaREM37-A1* construct into the Arabidopsis *atrem35* mutant, which is the closest homolog. For each construct, three independent lines, showing different levels of transgene expression were analyzed (**Supplementary Figure S6**). We included in our analysis *atrem34 35S:AtREM34* and *atrem35 35S:AtREM35* lines (Caselli et al., 2025), as controls.

**Figure 4:**
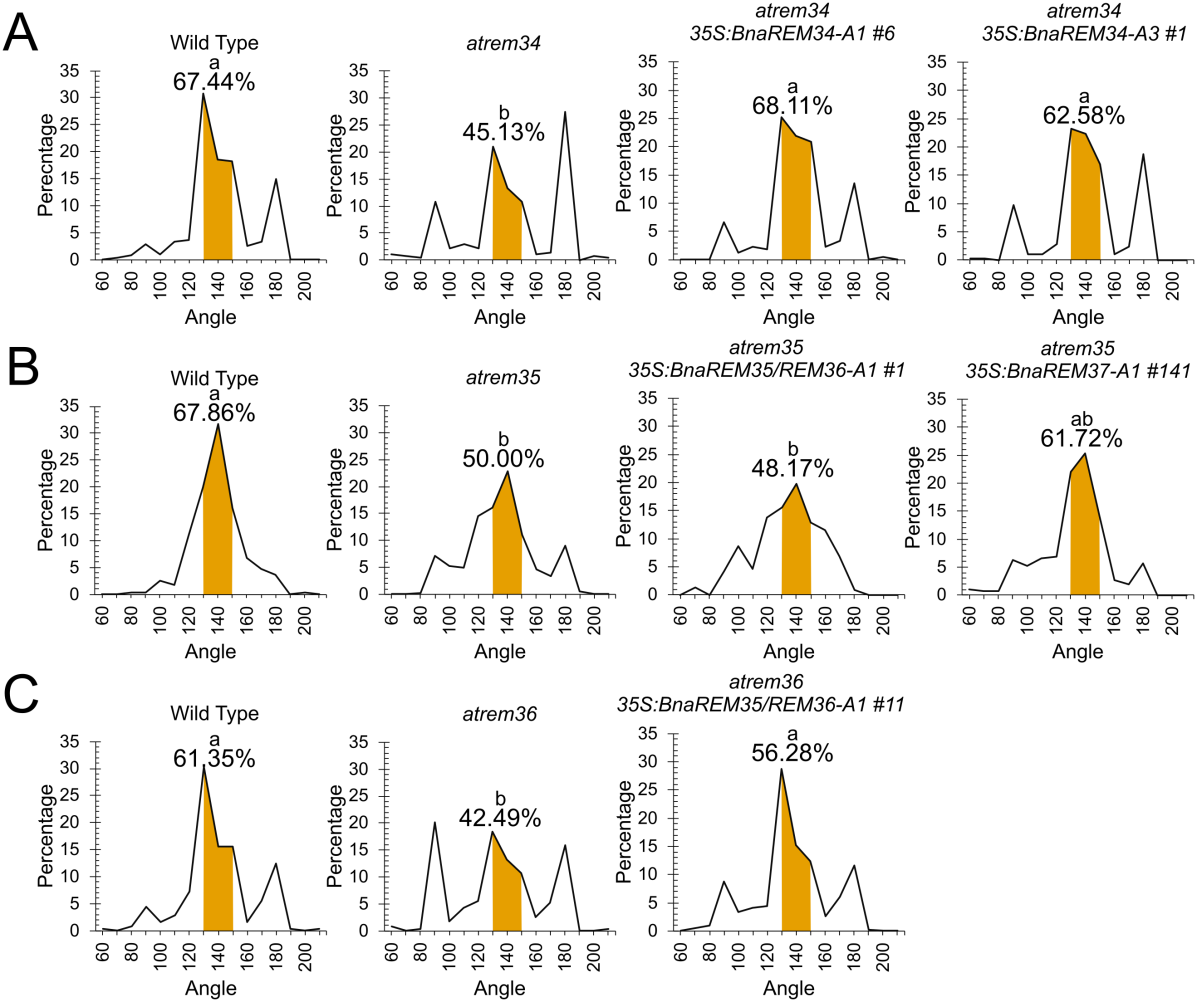
Phyllotaxis complementation tests. The graphs show the distribution of angles between two successive siliques. The canonical range 130°-150°, used to evaluate the complementation of the phyllotaxis phenotype is highlighted and the percentage of angles falling in that range is reported. The three single mutants, *atrem34, atrem35* and *atrem36*, and the wild type were used to assess the complementation ability of the *BnaREM* genes under study. **(A)***atrem34* complementation analysis. In wild type plants (12 plants, 390 angles), 67.44% of the angles between siliques fall into the canonical range, while in at*rem34* (13 plants, 277 angles) the same range accounts only for 45.13% of the measured angles. *atrem34* mutant plants overexpressing both *35S:BnaREM34-A1* and *35S:BnaREM34-A3* showed a complementation of the phyllotactic pattern, with a percentage of angles within the canonical range similar to the wild type. For each *BnaREM* gene, three independent transgenic lines were analyzed, one representative line is shown here. The three lines and the *atrem34 35S:AtREM34*, used as an additional control, are shown in **Supplementary Figure S6**. **(B)***atrem35* complementation analysis. In wild type plants (9 plants, 280 angles), 67.86% of the angles between siliques fall into the canonical rage, while in *atrem35* (10 plants, 324 angles) the same range accounts only for 50% of the measured angles; *atrem35* plants expressing *35S:BnaREM35/REM36-A1* showed a phenotype similar to *atrem35*, suggesting that this *B napus* gene cannot complement the Arabidopsis *rem35* mutant. On the other hand, in *atrem35* plants expressing *35S:BnaREM37-A1* we measured a partial rescue of the mutant phenotype. This indicates that *BnaREM37-A1* plays a role in phyllotactic patterning. Also in this case, three independent transgenic lines were analyzed and one representative line is shown here. The three lines and the *rem35 35S:AtREM35* overexpressing lines are shown in **Supplementary Figure S6**. **(C)***atrem36* complementation analysis, *atrem36* plants (9 plants, 233 angles) are characterized by 42.49% of angles between siliques falling into the canonical range, while in wild type (10 plants, 251 angles), this percentage was found to be 61.35%. In *atrem36* plants expressing *35S:BnaREM35/REM36-A1*, we observed a rescue of the phenotype, in contrast to what was observed for *atrem35.* This indicates that *BnaREM35/REM36-A1* was able to complement the *atrem36* loss-of-function mutant. Three independent transgenic lines were analyzed and one representative line is shown here. The analysis of all three lines is shown in **Supplementary Figure S6**. Statistical significance was assessed by Kruskal-Wallis and Dunn’s test in *atrem34* complementation and by ANOVA and Dunnett’s test for *atrem35* and *atrem36* complementation (p<0.05, tests according to the normality and homoscedasticity of the data). Different letters are given to significantly different genotypes.

In the lines expressing *BnaREM34-A1* (line 2: 68.54%; line 5: 67.74%; line 6: 68.11%) the range of angles falling into the 130° - 150° range was found to be similar to the one of the lines expressing *BnaREM34-A3* (line 1: 62.58%; line 9: 66.24%; line 11: 61.07%). In both cases, the percentages of canonical angles were close to the wild type value (67.47%), and significantly higher than those of the *atrem34* mutant (45.13%). The restoration of a wild type-like phenotype suggests a functional conservation between *AtREM34* and *BnaREM34-A1* and *BnaREM34-A3* (**Figure 4A**).

*BnaREM35/REM36-A1* was able to complement the Arabidopsis *atrem36* mutant but not *atrem35*. Specifically, the wild type exhibited a percentage of angles falling in the golden-angle range of 61.35%, while the *atrem36* mutant showed an almost 20% decrease of angles in the same range; the transgenic *atrem36 35S:BnaREM35/REM36-A1* lines 11, 110 and 115 displayed percentages of phyllotactic angles in the 130°-150° range of 56.28%; 60.35% and 56.44%, respectively, values statistically similar to wild type (**Figure 4C**). In contrast, the transgenic lines *atrem35 35S:BnaREM35/REM36A1* lines 1, 2 and 3 showed percentages of 48.17%, 51.61% and 36.76%, respectively, which is similar to the *atrem35* mutant, which has an average percentage of 50% (**Figure 4B**). These results indicate that *BnaREM35/REM36-A1* is functionally related to *AtREM36.*

Finally, the phenotype of the *atrem35 35S:BnaREM37-A1* lines suggests a functional conservation with *AtREM35*. Compared to percentage of siliques in the golden angles range of 67.86% measured in the wild type, and the 50% measured in the *atrem35*, two out of three transgenic lines (Lines 32 and 141) showed partial complementation, with 65.10% and 61.72% of siliques falling into the golden angle range, respectively, behaving similar to the *atrem35 35S:AtREM35* overexpression line (**Figure 4B**, **Supplementary Figure S6**).

Overall, these data support the notion of functional conservation across species and suggest that these genes in *B. napus* may play a role in phyllotaxis regulation.

### BnaREMs are able to homo- and heterodimerize

Many REM-REM protein complexes have been reported in Arabidopsis (Mendes et al., 2016; Caselli et al., 2019; Manrique et al., 2023) and it is known that AtREM35 can homodimerize and dimerize with AtREM34. To further characterize the BnaREM proteins and to explore whether there is functional conservation between these *B. napus* proteins and their Arabidopsis homologs, we investigated the conservation of their interaction profiles by using the yeast-2-hybrid (Y2H) assay.

These Y2H experiments showed that the BnaREM proteins under study were able to homo- and heterodimerize. Notably, comparing the interactions of the selected BnaREM proteins with their closest homologs in Arabidopsis, both conserved and divergent behavior was observed.

**Figure 5:**
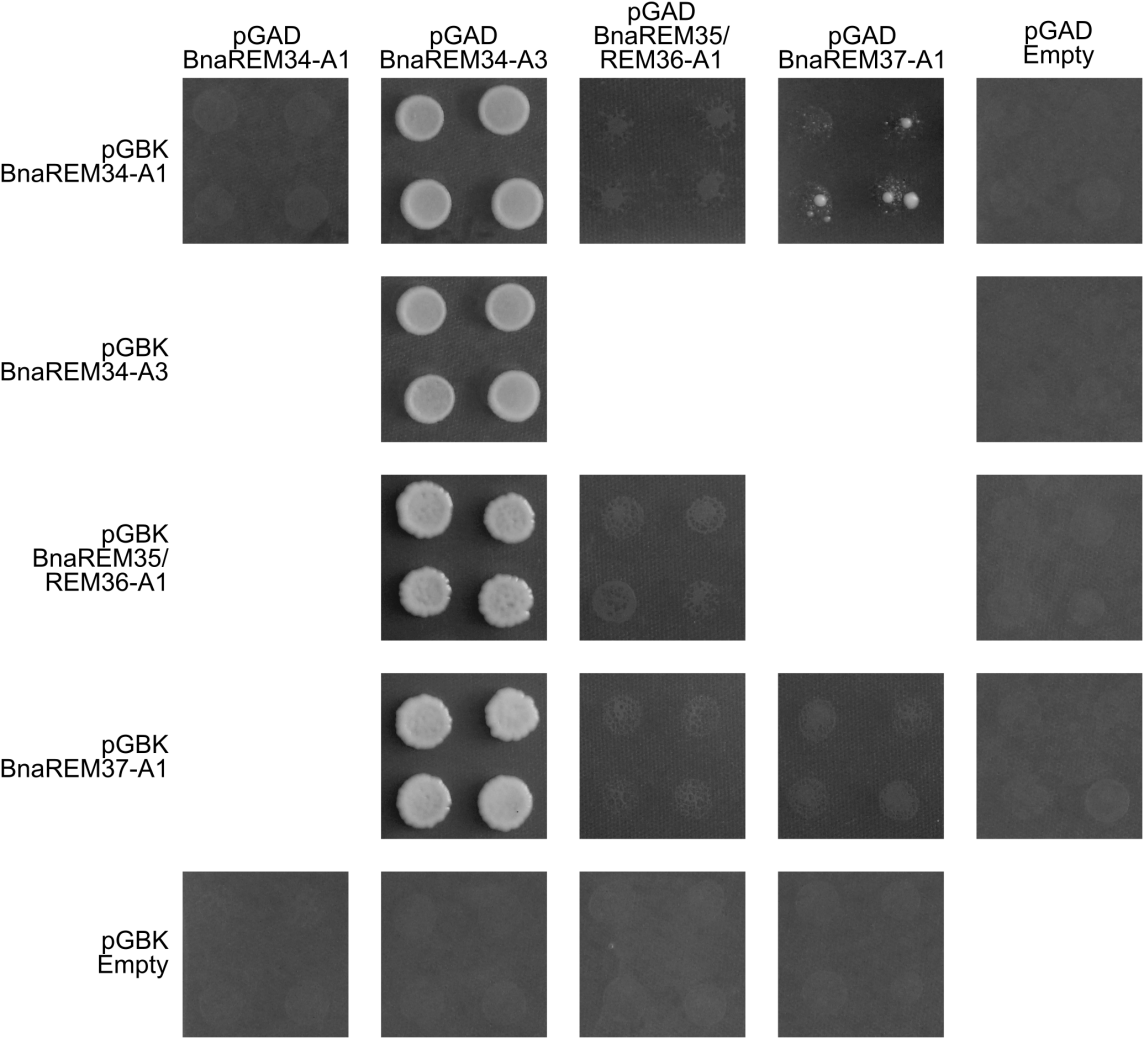
BnaREM homo and heterodimerizations. Y2H was employed to analyze the interaction ability of the selected BnaREM proteins. The CDSs of the proteins of interest were cloned in frame with the GAL4 Activation Domain (pGAD, Leu selection marker gene) or the GAL4 DNA Binding Domain (pGBK, Trp selection marker gene) and selective medium lacking Ade, His, Trp and Leu was used to test the interactions. AtREM35-AtREM35 and AtREM34-AtREM34 were used as positive and negative controls, respectively (**Supplementary Figure S7**). All the positive interactions were further confirmed by BiFC assay (**Supplementary Figure S7**).

Specifically, we found that BnaREM34-A3 could homodimerize and interact with all the selected BnaREM proteins (BnaREM34-A1, BnaREM35/REM36-A1, BnaREM37-A1). In contrast to AtREM34, BnaREM34A3 exhibited a high degree of promiscuity, displaying extensive interactions with all tested proteins, including itself (**Figure 5**).

BnaREM34-A1 and BnaREM35/REM36-A1 did not form heterodimers with each other, similar to the behavior of AtREM34 and AtREM36 in heterodimer formation (Caselli et al., 2019). Additionally, BnaREM35/REM36-A1 did not homodimerize (**Figure 5**), consistent with the interaction profile of AtREM36 (Caselli et al., 2019). These data suggest that BnaREM35/REM36-A1 is more closely related to AtREM36 than to AtREM35, which is coherent with the results obtained by the complementation assays.

BnaREM37-A1 showed a weak interaction with BnaREM34-A1 and a stronger one with BnaREM34-A3 (**Figure 5**). Comparing this observation with the results of the complementation assay supports functional similarity between BnaREM37-A1 and AtREM35.

Positive interactions identified through Y2H were all further validated in a plant context using Bimolecular Fluorescence Complementation (BiFC; **Supplementary Figure S7**). All the interactions revealed in yeast were confirmed in these *in planta* experiments, supporting the reliability of these Y2H interactions.

Overall, some of these interactions correspond to those observed in Arabidopsis, while others are novel, highlighting putative *B. napus* specific interactions.

### Link between BnaREMs and the auxin pathway

The AtREM35 protein, besides being able to homodimerize and heterodimerize with AtREM34, is also able to interact with two members of the Auxin Responsive Family, AtARF7 and AtARF19, which are also involved in phyllotaxis establishment (Caselli et al., 2025).

To investigate whether this interconnection between REMs and the auxin pathway is maintained in *Brassica napus*, the interactions between the BnaREMs and AtARF7 and AtARF19 were tested.

**Figure 6:**
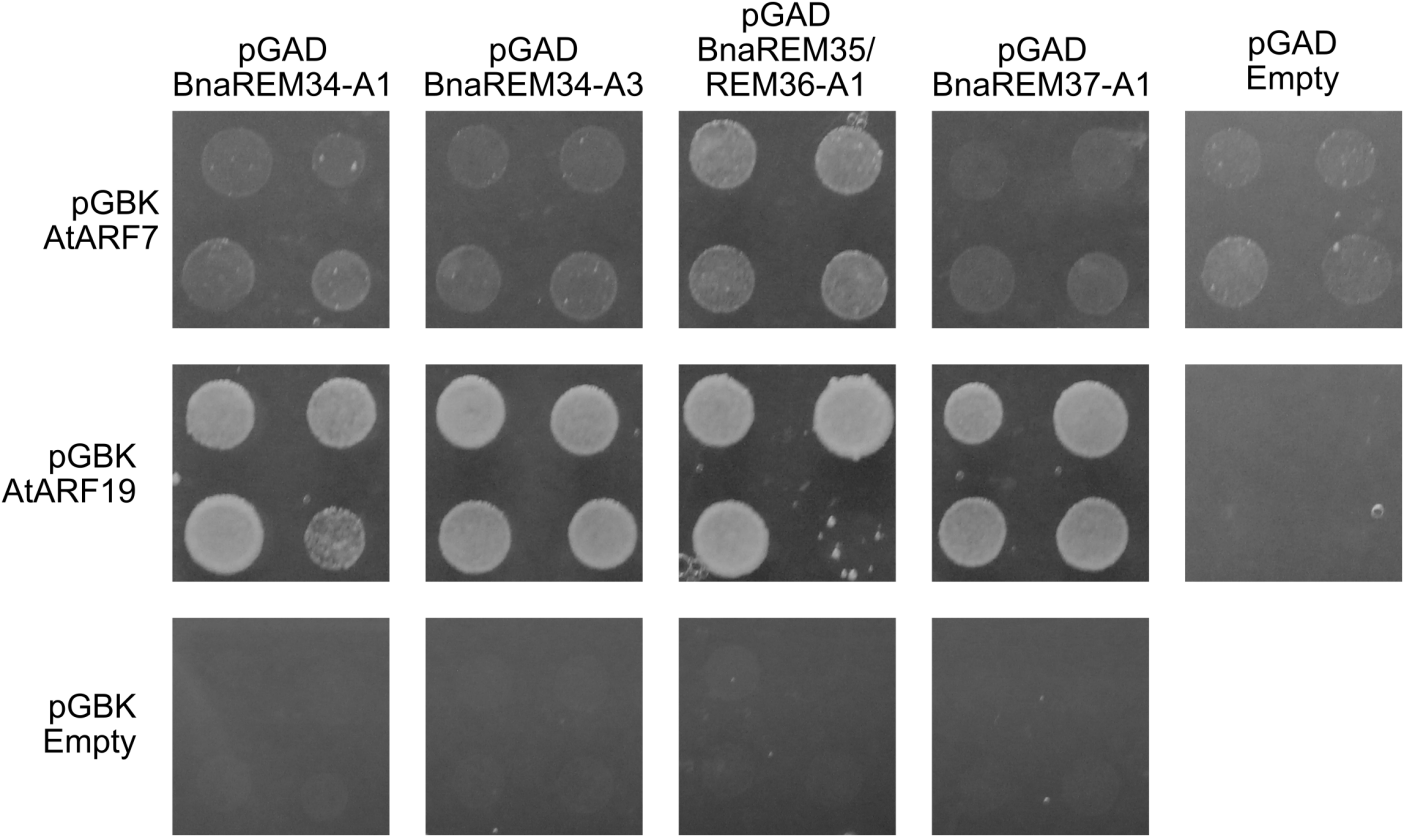
Y2H showing BnaREM-AtARFs interactions. The BnaREM CDS, cloned into the prey pGAD vector (GAL4 Activation Domain, Leu selection marker gene) were tested against AtARF7 and AtARF19, cloned in the bait pGBK vector (GAL4 DNA Binding Domain, Trp selection marker gene). Selective medium lacking Ade, His, Trp and Leu, supplemented with 100 mM of 3-At, was used to test the interactions. AtREM35-AtREM35 and AtREM34-AtREM34 were used respectively as positive and negative control (**Supplementary Figure S7**).

We found that BnaREM34-A1, BnaREM34-A3 and BnaREM37-A1 could only interact with AtARF19. BnaREM35/REM36-A1 instead could interact with both AtARF7 and AtARF19, retaining the same interaction profile of AtREM35. BnaREM37-A1, whose complementation data supported a role in phyllotaxis conserved with the one of AtREM35, was shown to interact with AtARF19 as AtREM35 does, but not with AtARF7 (**Figure 6**).

Our results suggest that a connection between the auxin pathway and the REM transcription factors also exists in *B. napus*, further strengthening the hypothesis that the *B. napus* REM genes are involved in establishing the correct phyllotaxis pattern in *B. napus.*

## Discussion

Feeding the world population without exacerbating humanity’s environmental impact stands as a paramount challenge of the century. Addressing the need to enhance food production without expanding agricultural land requires a focus on increasing crop yields. Phyllotaxis is an important determinant of the inflorescence architecture, which is closely linked to overall seed yield. Therefore, engineering of phyllotaxis/inflorescence architecture in seed crops like *Brassica napus* may provide a means to increase yield per hectare.

The analysis of the Arabidopsis *atrem34*, *atrem35* and *atrem36* double mutant combinations which we showed in this study, demonstrated that mutating multiple *rem* genes results in phyllotaxis changes and in a significant increase in siliques on the main stem. Importantly, the mutants didn’t show macroscopical developmental defects and maintained the same number of seeds per silique. This resulted in an overall increase in seed yield on the main stem of approximately 20% with respect to wild type plants.

We previously reported that *atrem35* and *atrem34 atrem35* are characterized by an enlarged IM. As traits such as phyllotactic alterations and increased IM size were already reported to be correlated with increments in siliques production (Bartrina et al., 2011; Sang et al., 2022; Zhang et al., 2022), engineering the *REM* genes might be promising to increase yield in agronomically relevant plants.

Building upon these findings, we investigated the functional conservation of these genes in the closely related and important seed crop *Brassica napus* by functional complementation experiments in Arabidopsis, aiming to explore the feasibility of translating our findings in the future to *B. napus*.

*Brassica napus* is the third oil crop in the world following palm and soybean, and it is the most important in Europe (FAOSTAT analytical brief 96, Agricultural production statistics 2010–2023; European Commission Directorate-General for Agriculture and Rural Development dataset, Oilseeds and protein crops production, 2025).

*B. napus* is mainly used for food processing, but is also used as an ingredient for personal care products, biofuel and industrial productions (such as bioplastics and others). Notably, around 15% of the global biodiesel production stems from rapeseed oil (OECD-FAO agricultural outlook 2021-2030). Increasing yield in rapeseed would therefore be of high value, serving both as a vital food source and as a resource for the bioeconomy.

The number of siliques, together with parameters as the number of seeds per silique and 1000-seed weight, determine the yield in *Brassica napus* (Yang et al., 2012). Furthermore, given the current high planting density of rapeseed, the yield of the main inflorescence has the most significant impact, contributing up to 73% of the whole plant yield (Yuan et al., 2022).

Consequently, identifying *B. napus REM* genes that share a conserved function with the *AtREM34*, *AtREM35* and *AtREM36* genes is of considerable interest.

The first requirement for the accurate identification of these *B. napus* REM genes is a deep bioinformatics analysis and critical reading of the results according to the genome and *REM* gene family evolution. *B. napus* is a recent allotetraploid, formed 7500 years ago from the interspecific hybridization between *Brassica rapa* and *Brassica oleracea* (Chalhoub et al., 2014). Its genome is composed of 38 chromosomes (2n, 1.2 Gb, Chalhoub et al., 2014; Chen et al., 2021), 20 of which are part of the A subgenome, (*B. rapa* derived), and 18 of the C subgenome (*B. oleracea* derived). Given the recent hybridization, its genome is highly redundant, even if some structural and functional interplay between the two subgenomes, incipits of gene losses and expression divergence, can be observed (Chalhoub et al., 2014).

In addition to the hybridization between *Brassica rapa* and *Brassica oleracea*, a whole genome triplication event is known to have occurred around 14–24 million years ago after the Arabidopsis– Brassica clade split (Bowers et al., 2003; Lysak et al., 2005; Wang et al., 2011). Therefore, for each gene in Arabidopsis six orthologs are theoretically expected in *Brassica napus*. This number can, in some cases, be reduced to an empirical average of four, because of random gene losses and a constitutive genome shrinkage for *Brassica rapa* (around 30%, Mun et al., 2009).

In the context of the research reported in this paper, the redundancy and complexity of the *B. napus* genome add up to the one of the *REM* family of transcription factors. Indeed, according to Romanel et al., 2009, 45 REM genes are present in the Arabidopsis genome. *AtREM34*, *AtREM35* and *AtREM36* belong to the subclass REM IX. This subclass contains 15 highly similar genes, probably derived from multiple events of gene duplication. Interestingly, most of the genes belonging to the REM IX class possess multiple B3 domains, a structure which again originated from tandem duplication events (Romanel et al., 2009). Within the REM IX subclass, 9 out of 15 genes are placed in tandem on chromosome 4 in the same cluster as *AtREM34, AtREM35* and *AtREM36*, while almost all the others are duplicated on chromosome 2.

From our analyses we identified six *B. napus* homologous genes for *AtREM34*, two genes which are related to both *AtREM35* and *AtREM36* and two genes which are homologous to *AtREM37*. The number of homologous genes we found in *B. napus* is consistent with all the above outlined evolutionary processes. Finally, the duplication of some of the *REM IX* subclass genes, including *AtREM35* and *AtREM36*, is supposed to be more recent than the Arabidopsis-Brassica clade split or to have happened in a similar period (Bowers et al., 2003; Lysak et al., 2005; Romanel et al., 2009; Wang et al., 2011). This might explain why we could not univocally assign a specific Arabidopsis homologous gene for *BnaREM35/REM36A1* and *BnaREM35/REM36C1*.

Taking into account genome evolution and expected gene redundancy, we decided to further expand our research including the homologs of *AtREM37*. Indeed, *AtREM37* is phylogenetically closely related to our genes of interest and placed together with them in the same gene cluster (Romanel et al., 2009, **Supplementary Figure S3**), which suggests a possible redundancy. Although *AtREM37* is not expressed in Arabidopsis (Mantegazza et al., 2014), we could not exclude that its homologs are expressed in *B. napus*.

To validate the expression of the identified *B. napus* genes, we performed both RT-qPCR and *in situ* hybridization. This showed that apart from the *BnaREM34-C2* gene (excluded from further analyses), all selected *B. napus REM* genes were expressed in the inflorescence and/or floral tissues, with a pattern that highly resembled the expression of *AtREM34, AtREM35* and *AtREM36* (Mantegazza et al., 2014; Caselli et al., 2019). This finding suggests a potential involvement of the *B. napus* genes in the determination of the phyllotaxis. Interestingly, by RT-qPCR, we also detected a lower but consistent expression of five *BnaREM34* genes in the SAM. This is consistent with what was previously reported in *Brassica oleracea* (Franco-Zorrilla et al., 1999) and in Arabidopsis (Franco-Zorrilla et al., 2002; Mantegazza et al., 2014; Manrique et al., 2023).

To further analyze the functional conservation of the selected candidate genes, we performed complementation analysis in the corresponding *rem* Arabidopsis mutants. Ectopic and specific expression of *Brassica* genes in Arabidopsis has already helped to infer the gene function in several studies (Yang et al., 2011; Zhang et al., 2017; Lyu et al., 2022; Yuan et al., 2022). Specifically, a complementation test can reveal the sub- and neofunctionalization of genes (Babula-Skowrońska et al., 2015; Schiessl et al., 2019), a common mechanism in species where multiple copies of the same ancestral gene are present. Therefore, it can be a valuable tool for selecting candidate genes for translation of know-how from model species to crops.

As the genes of the two parental subgenomes code for very similar proteins (**Supplementary Figure S4**) and the event of hybridization is recent, we performed this analysis on the candidates of the subgenome A, excluding *BnaREM34-A2* as it is highly similar to *BnaREM34-A1*. The results of the interspecific complementation test demonstrate a functional conservation between Arabidopsis and Brassica genes and further support the hypothesis of the involvement of *BnaREM* genes in phyllotaxis determination. Indeed, *BnaREM34-A1* and *BnaREM34-A3* could complement the mutated phyllotactic pattern of the *atrem34* mutant. *BnaREM35/REM36-A1*, on the other hand, could only complement *atrem36* and not *atrem35*. Despite the lack of a univocal assignment of an *AtREM* homolog in the phylogenetic analyses, these results suggest that *BnaREM35/REM36-A1* shares conserved functions with *AtREM36* rather than with *AtREM35*. *AtREM37* is not expressed in Arabidopsis; therefore, the mutant was never characterized. Notably, *BnaREM37-A1* could partially complement *atrem35*, disclosing a putative novel role for *REM37* in phyllotaxis establishment and indicating a functional conservation of *BnaREM37-A1* with *AtREM35*.

These data demonstrate that, as in Arabidopsis, the BnaREM transcription factors under study functionally diversified and suggest that multiple mutant combinations would have to be combined to alter the phyllotaxis for increased seed production.

To further explore the functional conservation of REM transcription factors, we investigated the conservation of REM protein dimerization in Arabidopsis and *B. napus* by yeast two-hybrid assays. REM protein interactions have been published in literature (Mendes et al., 2016; Caselli et al., 2019; Manrique et al., 2023) (Mendes et al., 2016; Caselli et al., 2019; Manrique et al., 2023), suggesting that this functional feature may be broadly conserved within the REM family. Notably, the *AtREM* genes on chromosome 4, except *AtREM39* and *AtREM40*, encode a specific additional domain, which defines a specific gene subgroup (Franco-Zorrilla et al., 2002). This domain is located at the C-terminus of the protein and is composed of hydrophobic heptads, which were predicted to mediate protein-protein interactions. Noteworthy, for AtREM34 and AtREM35 the ability of the domain to mediate protein interactions was experimentally confirmed (O’Shea et al., 1989; Franco-Zorrilla et al., 2002; Caselli et al., 2025). Additionally, since AtREM35 interacts with two members of the auxin signaling pathway, AtARF7 and AtARF19, we also investigated whether these interactions were conserved in Brassica. Our results demonstrate that the wide interaction capabilities of the REMs are conserved in *B. napus* and underline the possibility that a REM-ARF interactions can be involved in inflorescence development in *B. napus*, as recently reported in Arabidopsis (Caselli et al., 2025).

To conclude, this study underscores the significance of *Arabidopsis thaliana* as a model system for investigating crucial agronomic traits within the Brassicaceae family. We have identified candidate genes in *B. napus* whose silencing could potentially produce genotypes with altered phyllotaxis and increased seed yield. Therefore, applying CRISPR-Cas9 technology to target multiple *BnaREM* genes appears to be a promising strategy to increase seed yield per hectare in this important oilseed crop.

## Data Availability

The original contributions presented in the study are included in the article/**Supplementary Material**, further inquiries can be directed to the corresponding author/s.
