# Supplementary material for: REM genes controlling phyllotaxis and yield: bridging findings from Arabidopsis thaliana to Brassica napus
Source: Front Plant Sci. 2026 Feb 2;16:1743387. doi: 10.3389/fpls.2025.1743387 (PMC12907299; doi:10.3389/fpls.2025.1743387)

***REM* Genes Controlling Phyllotaxis and Yield:**

**Bridging Findings from *Arabidopsis thaliana* to *Brassica napus***

Carlotta C. Ferrario^1,#^, Francesca Caselli^1,#^, Shokhsanam Davlatboeva^1,3^, Evert-Jan Blom^2^,

Arjen van Tunen^2,4^, Martin M. Kater^1*,^ Veronica Gregis^1*^

^1^ University of Milan, Department of Biosciences, Via Celoria 26, 20133 Milan, Italy

^2^ Keygene Company, Agrobusiness Park 90, Wageningen, Gelderland: 6701 AA, Netherlands

^3^ Present address: Johannes Gutenberg-Universität Mainz, Saarstraße 21, 55122 Mainz, Germania

^4^ Present address: TunenAgroFood BV, Wageningen, Netherlands

^#^ These authors contribute equally to this work

*Equally contributing corresponding authors: [martin.kater@unimi.it](mailto:martin.kater@unimi.it) and [veronica.gregis@unimi.it](mailto:veronica.gregis@unimi.it)

ORCID:

Carlotta Ferrario: <https://orcid.org/0009-0007-6180-0115>

Francesca Caselli: <https://orcid.org/0000-0002-9375-6979>

Shokhsanam Davlatboeva: <https://orcid.org/0009-0006-0305-8293>

Martin Kater: <https://orcid.org/0000-0003-1155-2575>

Veronica Gregis <https://orcid.org/0000-0003-1876-9849>

**Supplementary Materials**

**Supplementary Figure S1.** *atrem36* mutant description

**Supplementary Figure S2.** *atrems* seed-set evaluation

**Supplementary Figure S3.** Phylogenetic tree of the REM XI subclade in Arabidopsis and *Brassica napus*

**Supplementary Figure S4.** BnaREMs and AtREMs protein similarity and homology

**Supplementary Figure S5.** *In situ* hybridization in flower organs and sense probes

**Supplementary Figure S6.** Complementation test

**Supplementary Figure S7.** Y2H controls and BiFC

**Supplementary Table S1.** Primers list

**
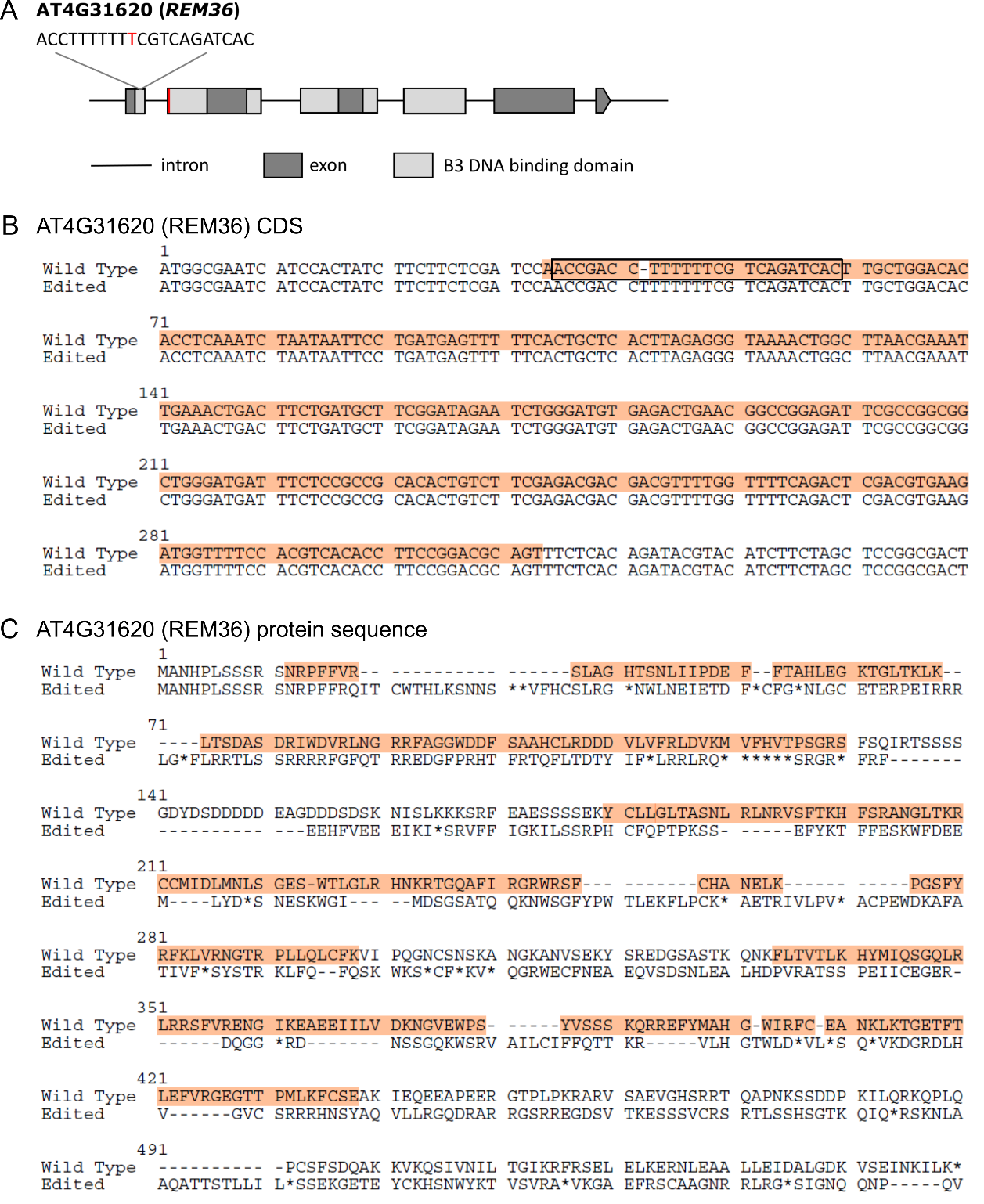
Supplementary Figure S1.** ***atrem36* mutant description** **(A)** Schematic representation of the structure of the *atrem36* mutant. Boxes: exons, lines: introns. The gene is characterized by three repetitions of the B3 DNA binding domain (light grey boxes). The sequence of the protospacer, which was designed on the first of the DNA binding domains, is shown. The genome editing resulted in a nucleotide insertion (red T), causing a frameshift and the premature formation of a stop codon (red line). **(B)** Alignment of REM36 wild type and edited CDS. The edited sequence is characterized by the insertion of a T at position 41; the protospacer is shown in a box and the first B3 domain sequence is highlighted. **(C)** The protein alignment between REM36 and *rem36*, the T insertion causes a frameshift starting from aa18 which leads to the formation of a stop codon in position 31, at the beginning of the first B3 DNA binding domain; the three B3 DNA binding domains are highlighted in the wild type sequence.


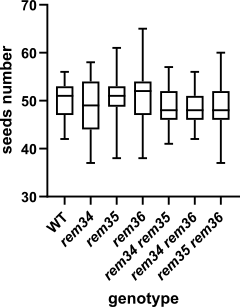
**Supplementary Figure S2.** ***atrems* seed-set evaluation.** Number of seeds per silique was evaluated in all the in *atrem* mutants. For each genotype, 5 siliques from 11 independent plants were analyzed. No significant differences were found compared to Wild Type (Kruskal Wallis – Dunn’s post hoc test).


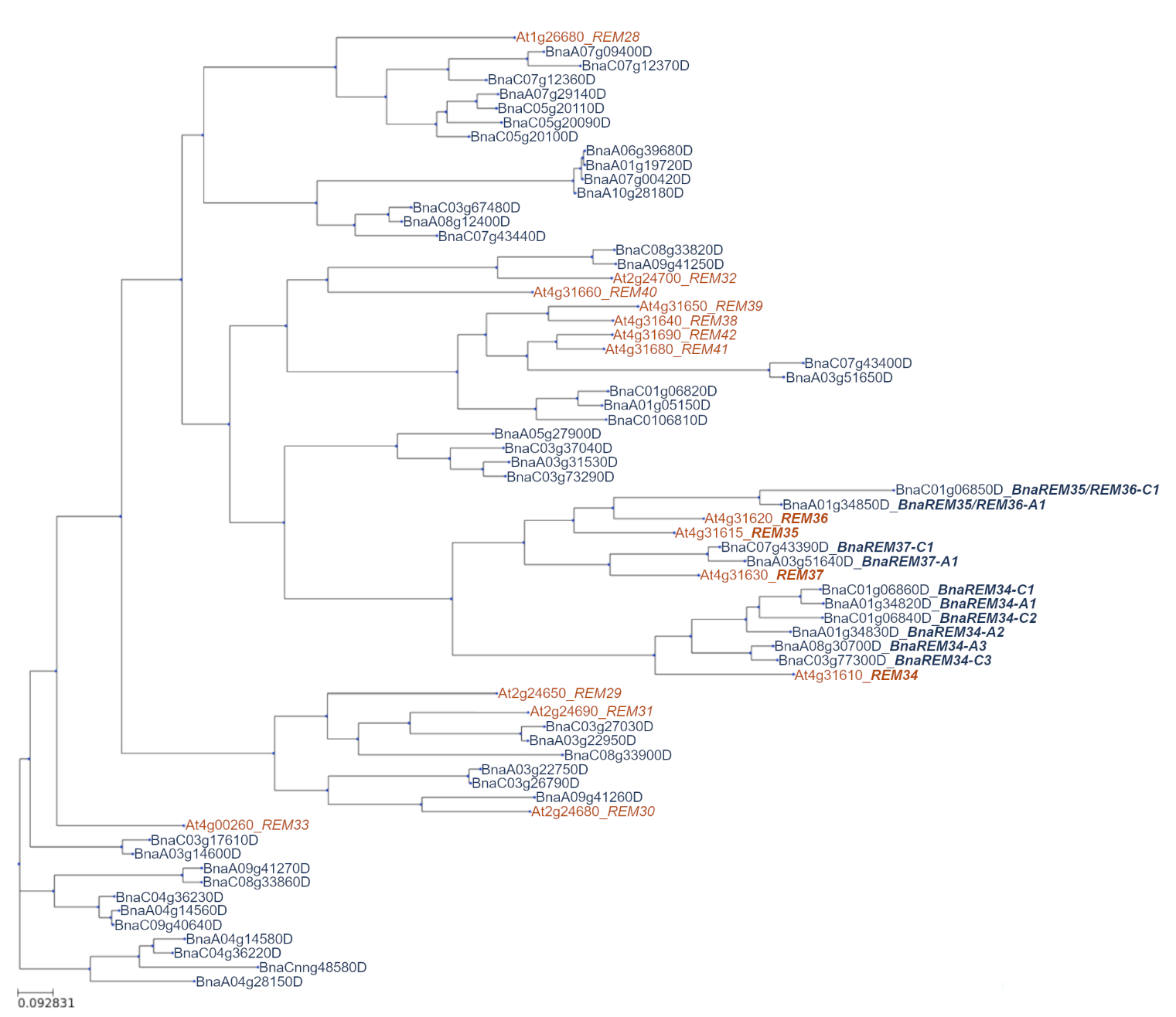
**Supplementary Figure S3.** Phylogenetic tree of the REM XI subclade in Arabidopsis and *Brassica napus.* Distance is shown as amino acid substitution per site. The *AtREM34, AtREM35, AtREM36* and *AtREM37,* as well as their Brassica homologues, are written in bold text.


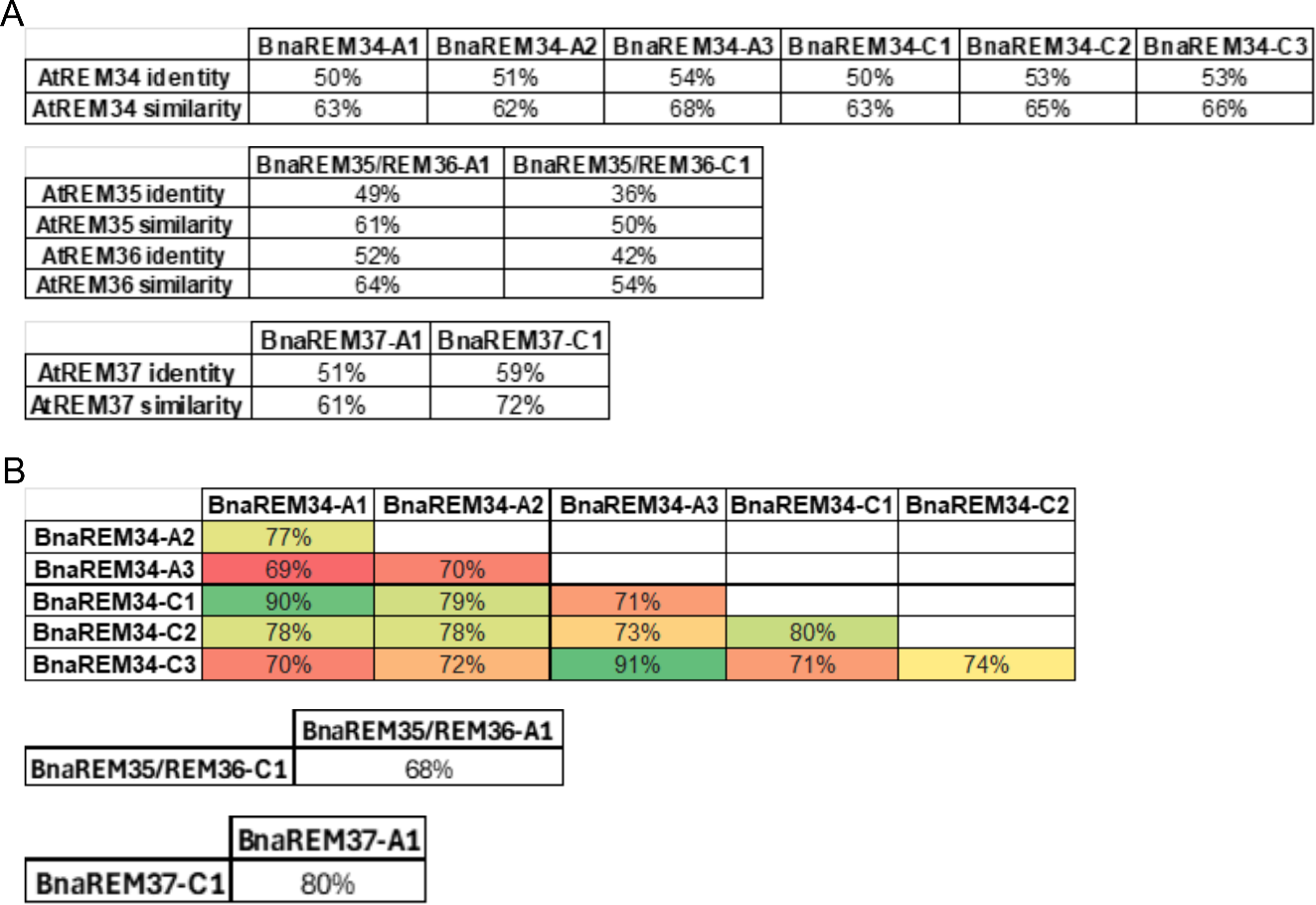
**Supplementary Figure S4.** BnaREMs and AtREMs protein similarity and homology. **(A)** Percentage of similarity and identity between the Arabidopsis and *Brassica napus* homologs. **(B)** Percentage of similarity between *Brassica napus* homologs. A color code is given to each row of the BnaREM34 homologs table to simplify the reading (green to red. Green = highest similarity. Red = lowest similarity.)


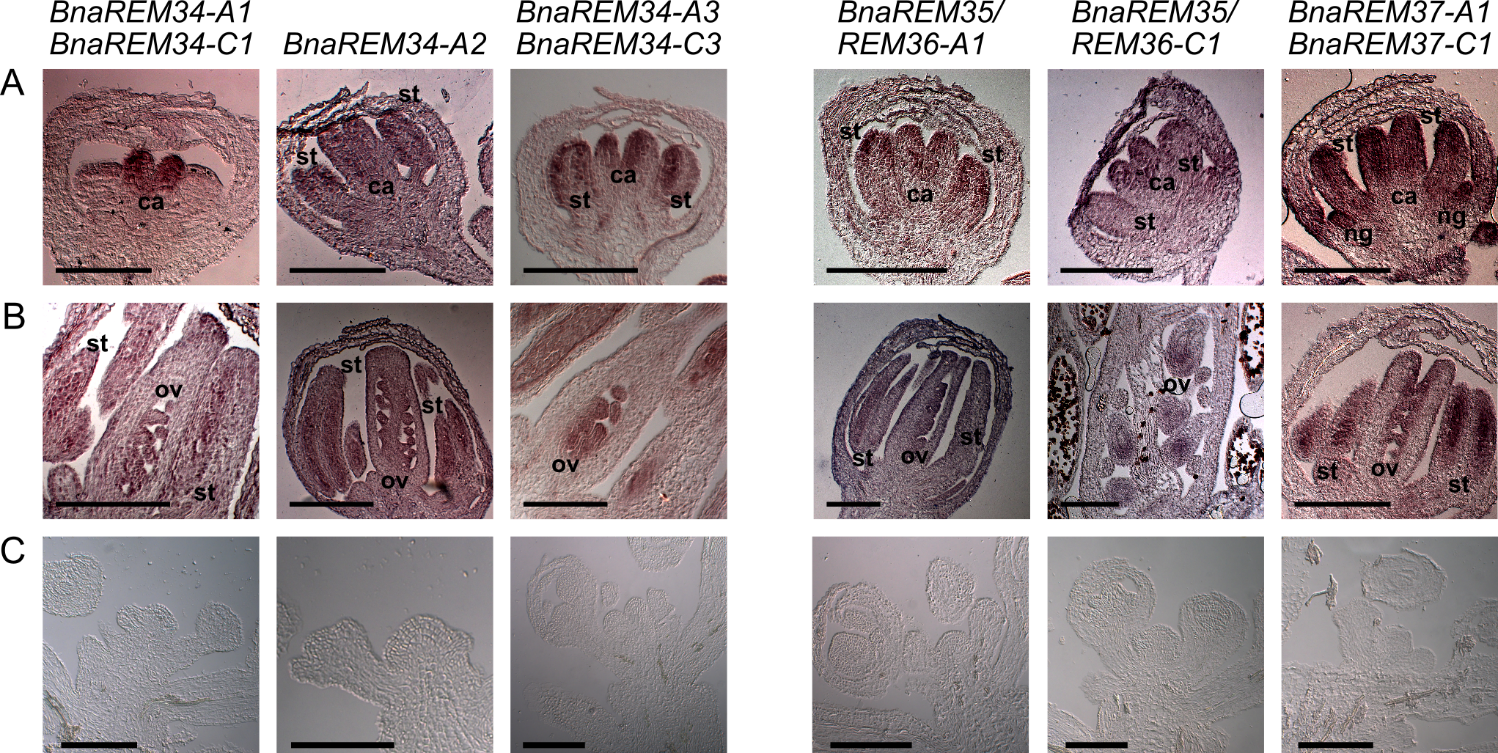
**Supplementary Figure S5. (A-B)** *In situ* hybridization in flower organs and sense probes. During flower development, *BnaREM34*, *BnaREM35/REM36* and *BnaREM37* expression is restricted in the inner floral whorls (carpel=ca; stamen=st) and nectary glands (ng) and is excluded from sepals. Later in development, the hybridization signal became clearly visible also in the ovules. Scale bar 200 µm. Sense probe controls. **(C)** The probe's specificity was assessed by performing *in situ* hybridization with sense probes on *Brassica napus* IM and floral organs, the same tissues employed for the expression pattern study. No signal was detected in the slides hybridized with the sense probes. Scale bar 200 µm.


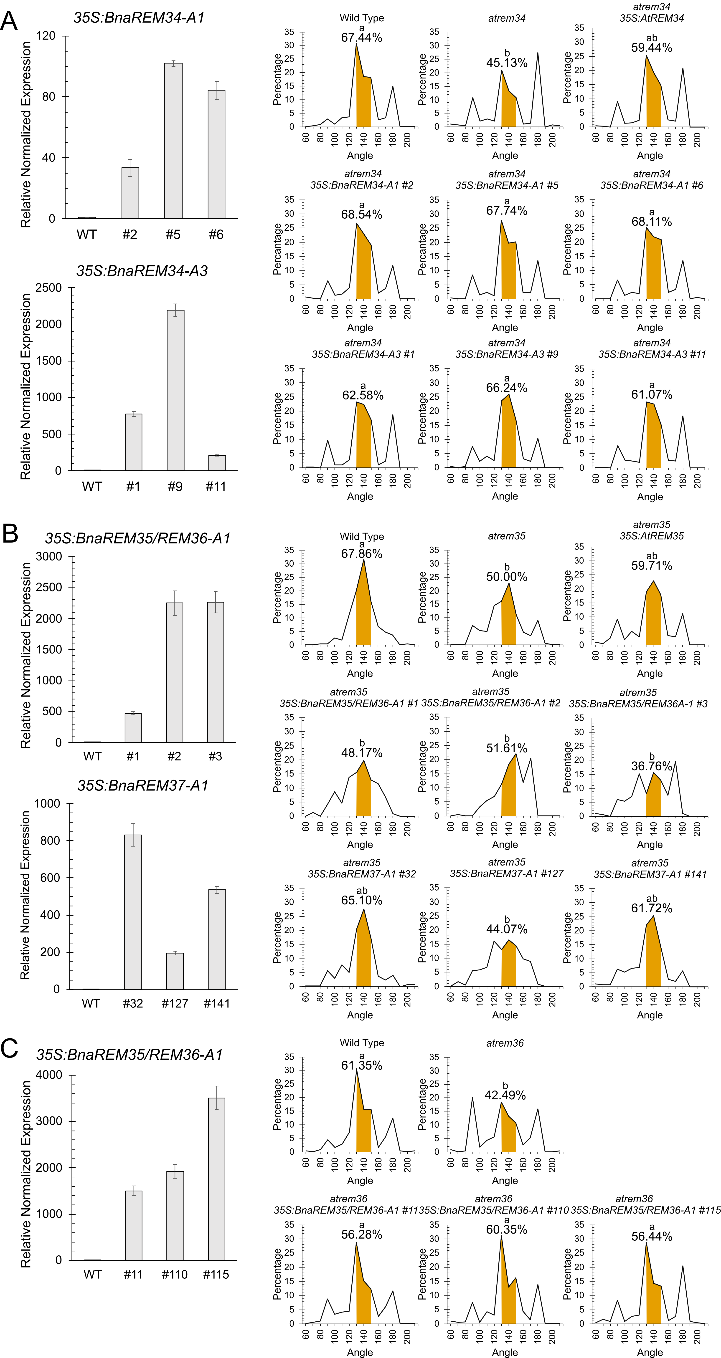
**Supplementary Figure S6.** Complementation test. **(A)** Left panel: Expression level of *35S:REM34-A1* and *35S:REM34-A3* in *atrem34* mutant background, expression was normalized on the *RCE1* transcript and the expression in the wild type was set to 1. Right panel: *atrem34* complementation analysis. For each *BnaREM* gene, three independent transgenic lines were analyzed and compared with both wild type and *atrem34.* The *atrem34 35S:AtREM34* was used as an additional control. **(B)** Left panel: Expression level of *35S:REM35/REM6-A1 a*nd *35S:REM37-A1* in *atrem35* mutant background, expression was normalized on the *RCE1* transcript and the expression in the wild type was set to 1. Right panel: *atrem35* complementation analysis. For each *BnaREM* gene, three independent transgenic lines were analyzed and compared with both wild type and *atrem35.* The *atrem35 35S:AtREM35* was used as an additional control. **(C)** Left panel: Expression level of *35S:REM35/REM6-A1* in *atrem36* mutant background, expression was normalized on the *RCE1* transcript and the expression in the wild type was set to 1. Right panel: *atrem36* complementation analysis. Three independent transgenic lines were analyzed and compared with both wild type and *atrem36.*

**Supplementary Figure S7.** Y2H controls and BiFC. **(A)** Y2H controls. Selective medium lacking Ade, His, Trp and Leu was used. AtREM35-AtREM35 and AtREM34-AtREM34 homodimers were respectively used as positive and negative controls. **(B)** BnaREM protein interaction analysis by BiFC. The experiment was used to confirm dimer formations revealed in Y2H analysis. The left line shows YFP (green) and Chl (blue), the right line includes the bright field too. p19K was co-infiltrated with the all the tested combinations. On the right panel the controls are depicted (AtREM34 homodimers: negative control, p19K only: negative control, AtREM35 homodimers: positive control). Scale bar=50 µm


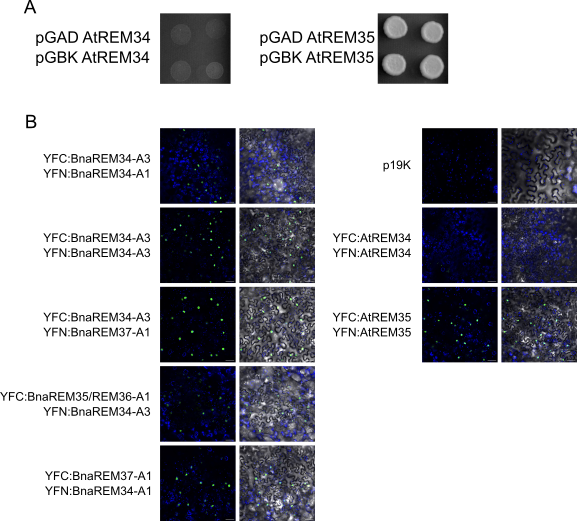

Supplement: Supplementary file 1 [file Table1.docx]
